# Improved Stability and Activity of a Marine Peptide-N6NH2 against *Edwardsiella tarda* and Its Preliminary Application in Fish

**DOI:** 10.3390/md18120650

**Published:** 2020-12-17

**Authors:** Huihui Han, Ting Li, Zhenlong Wang, Da Teng, Ruoyu Mao, Ya Hao, Na Yang, Xiumin Wang, Jianhua Wang

**Affiliations:** 1 Gene Engineering Laboratory, Feed Research Institute, Chinese Academy of Agricultural Sciences, 12 Zhongguancun Nandajie St., Haidian District, Beijing 100081, China; hanhuihui@caas.cn (H.H.); liting01@caas.cn (T.L.); wangzhenlong01@caas.cn (Z.W.); tengda@caas.cn (D.T.); maoruoyu@caas.cn (R.M.); haoya@caas.cn (Y.H.); yangna@caas.cn (N.Y.); 2 Key Laboratory of Feed Biotechnology, Ministry of Agriculture and Rural Affairs, Beijing 100081, China; 3 Chinese Herbal Medicine Laboratory, Feed Research Institute, Chinese Academy of Agricultural Sciences, Beijing 100081, China

**Keywords:** marine peptide, N6, N6NH2, antimicrobial activity, mechanism, *Edwardsiella tarda*, immune

## Abstract

*Edwardsiella tarda* can cause fatal gastro-/extraintestinal diseases in fish and humans. Overuse of antibiotics has led to antibiotic resistance and contamination in the environment, which highlights the need to find new antimicrobial agents. In this study, the marine peptide-N6 was amidated at its C-terminus to generate N6NH2. The antibacterial activity of N6 and N6NH2 against *E. tarda* was evaluated in vitro and in vivo; their stability, toxicity and mode of action were also determined. Minimal inhibitory concentrations (MICs) of N6 and N6NH2 against *E. tarda* were 1.29–3.2 μM. Both N6 and N6NH2 killed bacteria by destroying the cell membrane of *E. tarda* and binding to lipopolysaccharide (LPS) and genomic DNA. In contrast with N6, N6NH2 improved the stability toward trypsin, reduced hemolysis (by 0.19% at a concentration of 256 μg/mL) and enhanced the ability to penetrate the bacterial outer and inner membrane. In the model of fish peritonitis caused by *E. tarda*, superior to norfloxacin, N6NH2 improved the survival rate of fish, reduced the bacterial load on the organs, alleviated the organ injury and regulated the immunity of the liver and kidney. These data suggest that the marine peptide N6NH2 may be a candidate for novel antimicrobial agents against *E. tarda* infections.

## 1. Introduction

*Edwardsiella tarda* (*E. tarda*) is a Gram-negative bacterium with a wide range of hosts, including aquatic animals and humans [1]; it can cause serious systemic fish infections and high mortality deaths in seawater and freshwater, leading to extensive economic losses to the aquaculture industry [2,3,4]. Meanwhile, *E. tarda* is also a fatal pathogen in humans, causing bacteremia, gastrointestinal disease, empyema and endocarditis [5]. Recently, *E. tarda* has been a great threat to many important economic fish such as *Oreochromis niloticus* in China [6,7]. It has been reported that *E. tarda* is one of the most serious pathogens of tilapia, which heavily influences the healthy development of aquaculture [8,9,10]. At present, the control of *E. tarda* disease mainly depends on the use of antibiotics, resulting in aquatic environmental pollution and antibiotic resistance [4,11]. Therefore, it is crucial to find new antibacterial agents to combat *E. tarda*.

Host defense peptides (HDPs), also known as antimicrobial peptides (AMPs), are immunomodulatory molecules that have evolved to provide extensive protection against a variety of pathogenic bacteria [12]. Due to broad spectrum activity, rapid killing rate and low drug resistance potential, AMPs open an avenue for the development of antimicrobial agents [12,13]. The clearance of Gram-negative bacteria by AMPs may be an effective strategy to control and prevent the deterioration of drug resistance and lipopolysaccharide (LPS)-induced pathophysiological response [14,15]. Marine peptide NZ17074 (N1), a variant of arenicin-3 (Tyr5→Asn, Tyr17→His), isolated from marine invertebrate lugworm *Arenicola marina*, has potent antibacterial activity against Gram-negative bacteria and fungi, while is now in the preclinical stage [16,17,18]. However, N1 displays some toxicity towards eukaryotic cells. To improve activity and toxicity, N6 was generated by replacing Cys3 and Cys20 with Ala and then was aminated at the C-terminus, named N6NH2 [19,20]. Similarly to other amidated AMPs such as aurein and PMAP-23, N6NH2 can enhance antibacterial activity against *Salmonella typhimurium* and reduce cytotoxicity due to an increased cationic charge, which can promote their entry into bacterial cell membranes and reduce interactions with eukaryotic cells [20,21,22].

In this study, the antibacterial activity of N6 and N6NH2 against *E. tarda* was firstly evaluated in vitro; their properties (including stability, hemolysis, and cytotoxicity) and possible modes of action were further elucidated. Furthermore, therapeutic effects of N6NH2 were examined in a model of *O. niloticus* peritonitis induced by *E. tarda*.

## 2. Results

### 2.1. Identification and Susceptibility of the E. tarda Strain

The 16S rRNA (1431 bp) and *gyr*B (1357 bp) genes of *E. tarda* were amplified by PCR and confirmed by DNA sequencing. Blast analysis showed that both genes share 100% identity with both JN and HZHM strains of *E. tarda* (data not shown).

The disc diffusion method was used to analyze the resistance of *E. tarda* to 27 commonly used antibiotics. The results showed that *E. tarda* was sensitive to most antibiotics, but it developed resistance to amoxicillin, bacitracin, and furazolidone (Supplementary Table S2).

### 2.2. Properties, Structure, and Antimicrobial Activity of N6 and N6NH2

#### 2.2.1. Properties and Structure Analysis of Peptides

**Figure 1:**
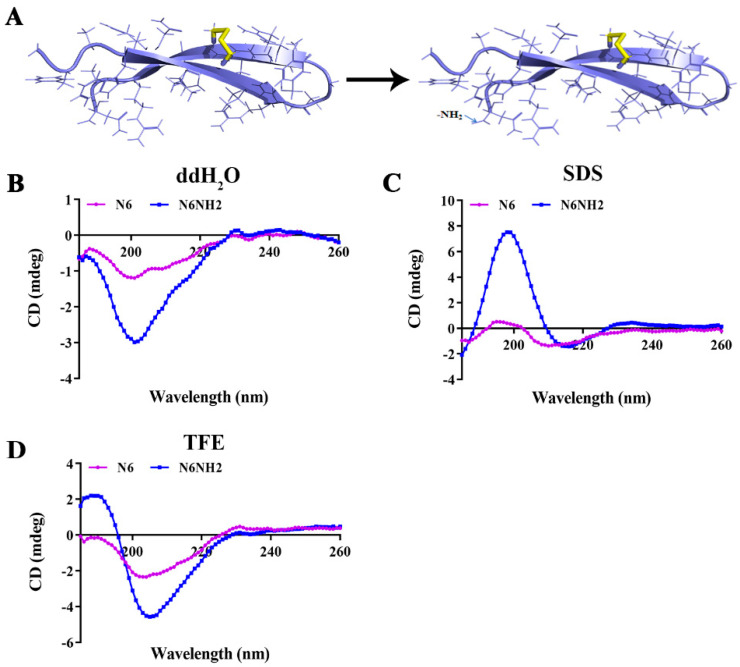
Structure and circular dichroism (CD) spectrum of N6 and N6NH2 in different solutions. (**A**) Structure of the peptides predicted by the I-TASSER server. (**B**–**D**) CD spectrum of the peptides in ddH_2_O (**B**), SDS (**C**), and TFE (**D**).

Both N6 and N6NH2 have similar properties including molecular weight (MW), hydrophobicity, instability index (II) and aliphatic index (AI). However, N6NH2 has more positive charges and a higher isoelectric point (PI) than N6 (Table 1). The structure analysis showed that N6 and N6NH2 have one rigid disulfide bridge Cys7-Cys16, which links the two β-strands (Cys3-Asn10 and Arg13-Cys20) and forms an anti-parallel β-sheet (Figure 1A); there is no structural difference between N6 and N6NH2.

The secondary structure of peptides in different solutions was measured by circular dichroism (CD) spectroscopy; ddH_2_O, sodium dodecyl sulfate (SDS) and trifluoroethanol (TFE) simulate a watery, hydrophobic and membrane-like environment [23]. The secondary structures of N6NH2 and N6 in ddH_2_O were characterized by a coil and antiparallel strand or β-turn with a negative minimum at 200 nm (Figure 1B). N6NH2 showed a more significant increase in α-helix and antiparallel strand in SDS solution than N6 (Figure 1C). Similarly, the CD spectrum of N6 and N6NH2 in 50% TFE showed one negative dichroic band at approximately 205 nm, and the positive maximum at 180 nm (strong) and at 230 nm (weak) (Figure 1D), indicating that β-turn, coil and antiparallel sheet are the main structures of N6 and N6NH2.

#### 2.2.2. Minimum Inhibitory Concentration (MIC), Minimum Bactericidal Concentration (MBC) and Killing Curve

The MIC and MBC values of peptides against Gram-positive and Gram-negative bacteria were measured to detect their antimicrobial activity. As shown in Table 2, the MICs of N6NH2 against *E. tarda*, *Aeromonas veronii* and *Staphylococcus hyicus* were 3.2, 1.6, and 3.2–12.8 μg/mL, respectively, lower than N6 (MICs of 6.4, 6.4, and 16–64 μg/mL, respectively) and norfloxacin (MICs of 0.5, 2, and 4–16 μg/mL, respectively), indicating its more potent antibacterial activity than N6 and norfloxacin. However, the activity of N6NH2 against other Gram-negative bacteria such as *Escherichia coli*, *S. typhimurium*, *S. enteritidis* and *S. pullorum* (MICs of 0.8–25.6 μg/mL) was lower than that of N6 (MICs of 0.25–4 μg/mL) and norfloxacin (0.06–1 μg/mL). Furthermore, N6NH2 displayed higher antibacterial activity against Gram-negative bacteria (except *Pseudomonas aeruginosa* CICC21630), with MICs of 0.8–3.2 μg/mL, than against Gram-positive bacteria such as *S. aureus* and *S. hyicus,* with MICs of 3.2–12.8 μg/mL. The MBCs of N6, N6NH2 and norfloxacin against *E. tarda* were 12.8, 6.4, and 2 μg/mL, respectively (data not shown).

**Figure 2:**
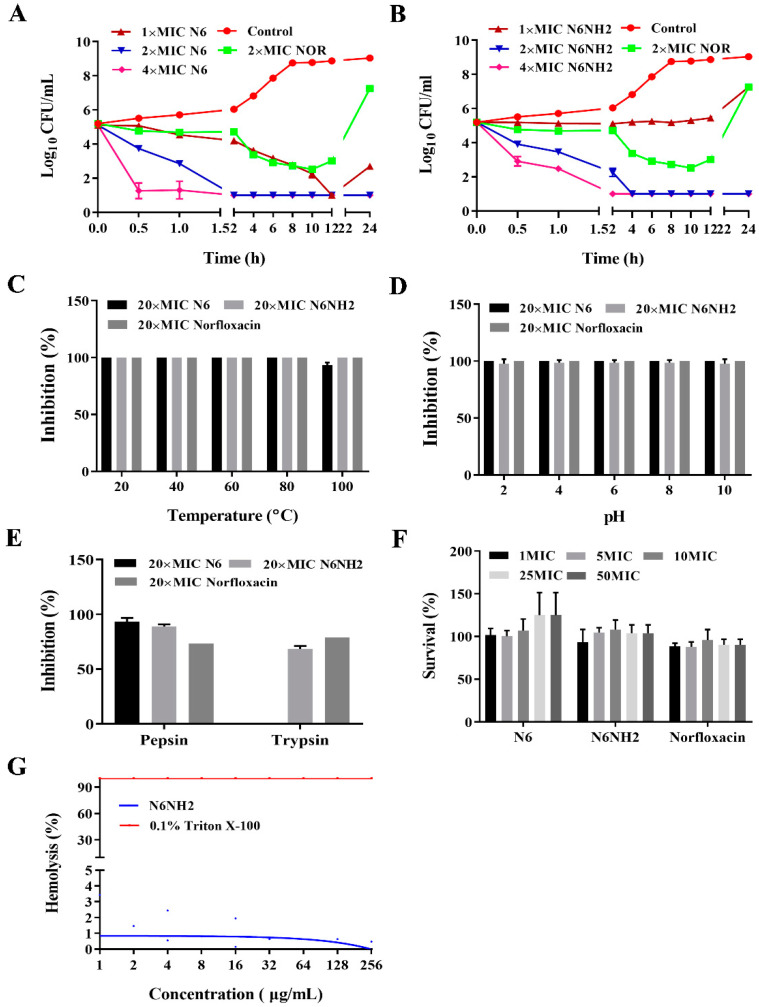
Killing curves, stability, cytotoxicity and hemolytic activity of N6 and N6NH2. (**A**,**B**) Killing curves of N6 (**A**), N6NH2 (**B**) or norfloxacin against *E. tarda.* Bacterial cells were treated with 1 ×, 2 × and 4 × MIC N6 or N6NH2, and bacteria were counted on the plate. (**C**–**E**) The effects of temperature (**C**), pH (**D**) and proteases (**E**) on the antibacterial activity of N6, N6NH2 or norfloxacin against *E. tarda*. The final concentrations of N6, N6NH2 and norfloxacin were 128 μg/mL, 64 μg/mL and 10 μg/mL, respectively. (**F**) Cytotoxicity of the peptides and antibiotic against RAW 264.7 cells. (**G**) Hemolytic activity of N6NH2 against murine erythrocytes. The results are given as the mean ± SEM (*n* = 3).

The time–kill kinetic curves are shown in Figure 2A; 1 × MIC N6 killed almost all the bacteria at 12 h, and 2 × and 4 × MIC of N6 could kill all bacteria within 2 h, which is more than norfloxacin. In contrast, 2 × and 4 × MIC N6NH2 killed the bacteria at 4 h and 2 h, respectively, but 1 × MIC N6NH2 did not inhibit bacteria (Figure 2B).

### 2.3. High Stability of N6 and N6NH2 to Temperature, pH, and Proteases

The antimicrobial activity of N6NH2 and norfloxacin against *E. tarda* did not change when exposed to 20, 40, 60, 80 or even 100 °C, but the activity of N6 decreased by 6.7% at 100 °C (Figure 2C). This may be associated with the C-terminal amidation of N6, enhancing the thermal tolerance and antibacterial activity [24]. Both N6 and norfloxacin exhibited strong antibacterial activity at different pH values of 2–10, but the activity of N6NH2 was slightly reduced by 2.3%, 1.3%, 1.3%, 1.3% and 2.3% at pH 2, 4, 6, 8, and 10, respectively (Figure 2D). Both N6 and N6NH2 were resistant to pepsin; however, N6NH2 was more resistant to trypsin than N6 (Figure 2E).

### 2.4. No/Low Cytotoxicity and Hemolysis of N6 and N6NH2

The 3-(4,5-dimethylthiazol-2-yl)-2,5-diphenyltetrazolium bromide (MTT) result showed that N6 and N6NH2 had very low cytotoxicity to mouse peritoneal RAW 264.7 macrophages (cell survival rate >93.35%), but norfloxacin showed some toxicity, with a cell survival rate of 73% (Figure 2F). Meanwhile, the hemolysis of N6NH2 towards mouse erythrocytes was 0.19% at a concentration of 256 μg/mL, lower than that of N6 (1.9%) [19], indicating their low hemolytic activity (Figure 2G).

### 2.5. Mechanism of N6 and N6NH2 against E. tarda

#### 2.5.1. Peptides Permeabilized the Outer Membrane of *E. tarda*

**Figure 3:**
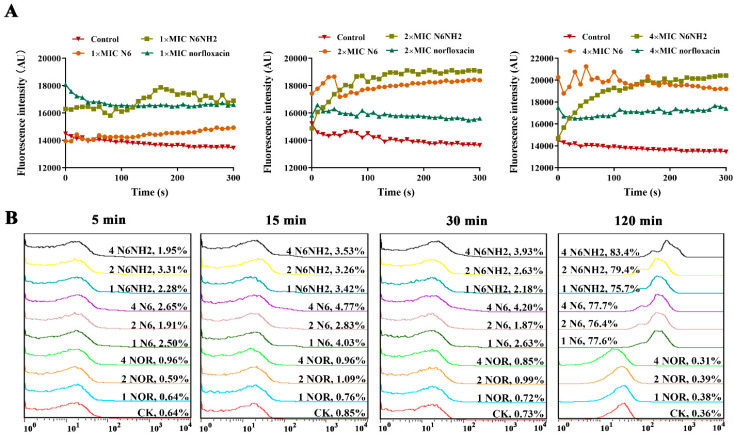
Effects of N6 and N6NH2 on the cell membrane of *E. tarda*. *E. tarda* cells were treated with 1×, 2× and 4× MIC N6, N6NH2 or norfloxacin and detected by a microplate reader and flow cytometer. (**A**) Effects of peptides on the outer membrane. (**B**) Effects of peptides on the inner membrane.

The ability of peptides to permeabilize the outer membrane was determined by *N*-phenyl-1-naphthylamine (NPN) fluorescent dye. When NPN enters the cell, the outer membrane is disturbed and fluorescence intensity increases. As shown in Figure 3A, both N6 and N6NH2 rapidly permeabilized the outer membrane of *E. tarda* within 1 min, and higher concentrations of peptides resulted in a stronger NPN signal (15,000–20,000), which was contrary to norfloxacin (18,000–16,000). This suggests that both N6 and N6NH2 induce a time- and concentration-dependent NPN fluorescence increase in *E. tarda* cells and that the permeabilization capacity of N6NH2 is higher than that of N6 (Figure 3A).

#### 2.5.2. Peptides Penetrated the Inner Membrane of *E. tarda*

Red fluorescent dye propidium iodide (PI) is blocked outside the intact cell membrane but can penetrate the damaged cell membrane and insert nucleic acids. PI fluorescence intensity indicates the level of cell membrane integrity. As shown in Figure 3B, in the untreated and norfloxacin-treated control, the percentage of *E. tarda* stained with PI was less than 1%, indicating an intact cell inner membrane. After treatment with N6 and N6NH2, the percentage of *E. tarda* stained with PI was less than 5%, indicating that the peptides hardly penetrated the bacterial inner membrane within 30 min (Figure 3B). However, after treatment with N6 and N6NH2 for 2 h, the percentages of PI-permeable *E. tarda* cells were 77.7% and 83.4%, respectively, indicating that N6NH2 has a stronger ability to penetrate the inner membrane than N6.

#### 2.5.3. Peptides Bound to Genomic DNA

**Figure 4:**
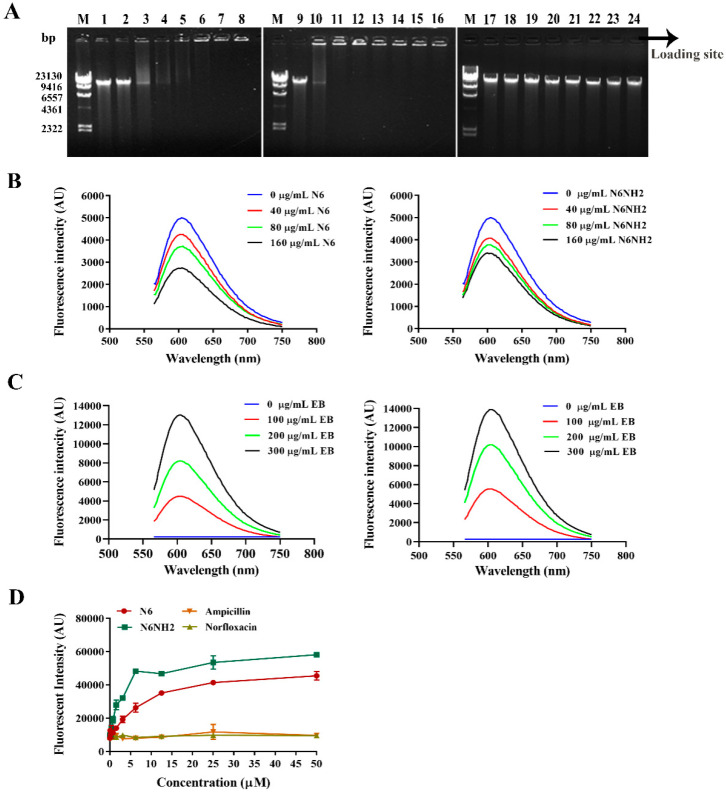
Interaction of N6 and N6NH2 with bacterial DNA and LPS. (**A**) Interaction of N6, N6NH2 (0–1000 μg/mL) or norfloxacin with bacterial genomic DNA (0.25 μg) according to analysis of the electrophoretic mobility of DNA. M: DNA marker λDNA/HindIII; 1–8: the mass ratios of N6/DNA were 0, 0.5, 1, 2, 4, 6, 8, and 10, respectively; 9–16: the mass ratios of N6NH2/DNA were 0, 0.5, 1, 2, 4, 6, 8, and 10, respectively; 17–24: the mass ratios of norfloxacin/DNA were 0, 0.5, 1, 2, 4, 6, 8, and 10, respectively. A black arrow indicates the location of loading sites. (**B**) Competitive binding of N6 (left) or N6NH2 (right) and EB with bacterial genomic DNA. A fixed amount of bacterial DNA (10 μg)-EB (0.15 μg) was treated with increasing amounts of peptides (the concentrations of 0, 40, 80 and 160 μg/mL). (**C**) Fluorescence spectra of DNA-N6 (left) and DNA-N6NH2 (right) in the presence of increasing amounts of EB. A fixed concentration of DNA (5 μg)-peptide (8 μg) was treated with increasing concentrations of EB (0, 100, 200 and 300 μg/mL, 1.5 μL). (**D**) Interaction of N6, N6NH2 or norfloxacin with LPS (ability of LPS to displace a peptide-bound BC probe).

To explore potential intracellular targets of peptides, the DNA binding properties of N6 and N6NH2 were investigated by analyzing the electrophoretic mobility of DNA. As shown in Figure 4A, with the increasing amounts of peptides, the migration of bacterial genomic DNA through the gel decreased. N6 inhibited the migration of *E. tarda* genome DNA at a mass ratio of peptide and DNA greater than 1. N6NH2 could disturb the migration of the genomic DNA from *E. tarda* within the mass ratio ranges from 0.5 to 10. Moreover, with increasing amounts of DNA, the migration of bacterial genomic DNA through the gel increased (Supplementary Figure S1). Additionally, both peptides could bind to bacterial DNA in a concentration-dependent manner. However, for norfloxacin, no DNA migration was observed at a mass ratio of 10. This result indicates that N6NH2 has a stronger binding capacity than N6 (Figure 4A); it may be attributed to increased net charges, which facilitate the electrostatic interaction between the peptide and the polyanions [22].

Additionally, both N6 and N6NH2 prevented the migration of fish genome DNA at a mass ratio of 2 and 1, respectively (Supplementary Figure S2), indicating some toxicity of peptides at a high concentration of peptides. Thus, effective dosage and toxicity of peptides should be weighed properly when they are administered to animals.

#### 2.5.4. Peptides Competitively Bound Genomic DNA with Ethidium Bromide (EB)

The fluorescence intensity of free EB is low, but after binding to DNA, it can emit strong fluorescence due to the insertion between adjacent base pairs in the double helix structure of DNA. It coexists with reagent molecules, and when performing similar reactions, the enhanced fluorescence can also be quenched; this can be used to detect the patterns of peptide binding to DNA [25]. As shown in Figure 4B, when different concentrations of peptides were added into a certain amount of DNA–EB solution (10 μg DNA/sample), with the increase of peptide concentrations, the fluorescence intensity of DNA-EB decreased significantly accompanied by a small blue shift, indicating that N6 and N6NH2 can compete with EB in binding to *E. tarda* genomic DNA in a concentration-dependent manner, and some EB molecules previously inserted into DNA bases may be replaced by N6 and N6NH2. However, when the concentration reached 160 μg/mL, the binding affinity of N6 was stronger than that of N6NH2.

Further support for peptide binding to DNA via intercalation was given through competitive binding assay of EB with bacterial DNA against N6 or N6NH2. With the addition of EB, the fluorescence of the DNA–peptide complex increased gradually (Figure 4C). Fluorescence of the DNA–N6NH2 complex was higher than that of DNA-N6, indicating that some of the EB molecules can intercalate into the DNA base pairs instead of peptides and that N6NH2 has a more potent ability to bind to DNA than N6.

#### 2.5.5. Peptides Bound to Bacterial LPS

LPS is a key component of outer leaflets of Gram-negative bacteria, which can protect bacteria from invasion by various host defenses. To further reveal the mechanism of action of N6 and N6NH2, the BODIPY’-TR-cadaverine (BC) replacement method was used to evaluate the ability of peptides to bind to LPS. As shown in Figure 4D, neither ampicillin nor norfloxacin replaced the BC probe or bound to LPS; N6 and N6NH2 had potent LPS-binding affinity in a concentration-dependent manner, indicating that the binding capacity of N6NH2 is higher than that of N6 (Figure 4D).

### 2.6. Effects of N6 and N6NH2 on Morphology of E. tarda

**Figure 5:**
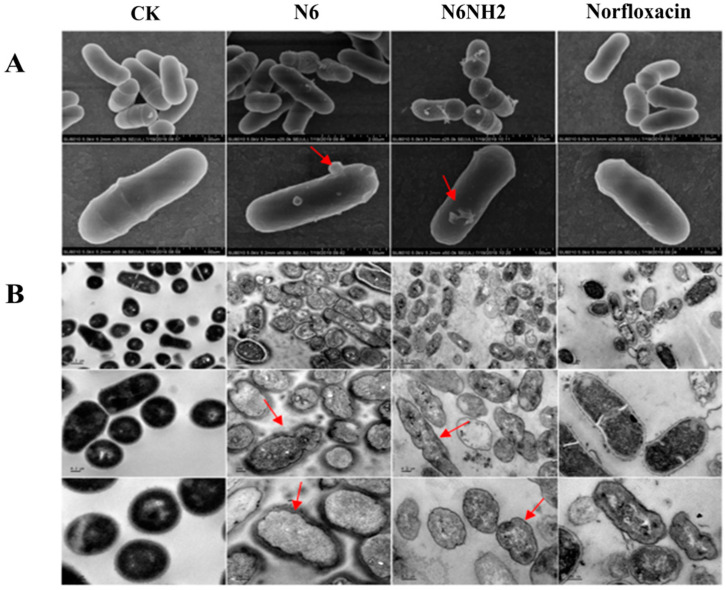
Effects of N6 and N6NH2 on the cell morphology and ultra-structures of *E. tarda.* Bacteria in mid-logarithmic growth phases were treated with peptides or antibiotic at 4 × MIC for 2 h. (**A**) SEM images of *E. tarda* cells treated with N6, N6NH2 or norfloxacin. (**B**) TEM images of *E. tarda* cells treated with N6, N6NH2 or norfloxacin.

*E. tarda* cells were treated with 4 × MIC N6, N6NH2 or norfloxacin for 2 h, and morphologic changes of cells were monitored by scanning electron microscopy (SEM). As shown in Figure 5A, the untreated cells showed a completely smooth surface. After treatment with N6 and N6NH2, protrusions and filamentous substances were observed on the cell surface. In the norfloxacin-treated group, there were no significant changes in the surface morphology and structure of bacterial cells (Figure 5A).

Transmission electron microscopy (TEM) was used to observe the effects of N6 and N6NH2 on the ultrastructure of *E. tarda* cells. As shown in Figure 5B, in the untreated control, normal morphology, intact cell membrane and uniform cytoplasmic electron density were observed in the cells. After exposure to 4 × MIC N6 and N6NH2 for 2 h, over 95% of abnormal cells were observed; this appeared to change cell morphology, shrinkage of the outer membrane, disappearance of the inner membrane, leakage of intracellular contents and heterogeneous electron density in the cytoplasm. However, the cells were not significantly affected by norfloxacin (Figure 5B). This suggests the membrane-disruptive potential of N6 and N6NH2.

### 2.7. N6NH2 Protected O. niloticus from Lethal Challenge with E. tarda

#### 2.7.1. Median Lethal Concentration (LC_50_) and Minimum Lethal Concentration (LC_100_) Values of *E. tarda*

Due to a better bactericidal effect of N6NH2 than N6, N6NH2 was chosen to further confirm the application potential in the *O. niloticus* peritonitis model of *E. tarda* infection. According to the relationship between the mortality rate of fish and the *E. tarda*-inoculated concentration, the LC_50_ value of *E. tarda* calculated by linear interpolation was 2.5 × 10^7^ CFU/mL. The LC_100_ value of *E. tarda* was 1 × 10^9^ CFU/mL.

#### 2.7.2. Protection of Fish from Infection with *E. tarda*

After intraperitoneal injection with LC_100_ of *E. tarda* (1.0 × 10^9^ CFU/mL), *O. niloticus* was treated with intraperitoneal injection of different concentrations of peptides or antibiotics for one-shot protection (6 fish/group).

**Figure 6:**
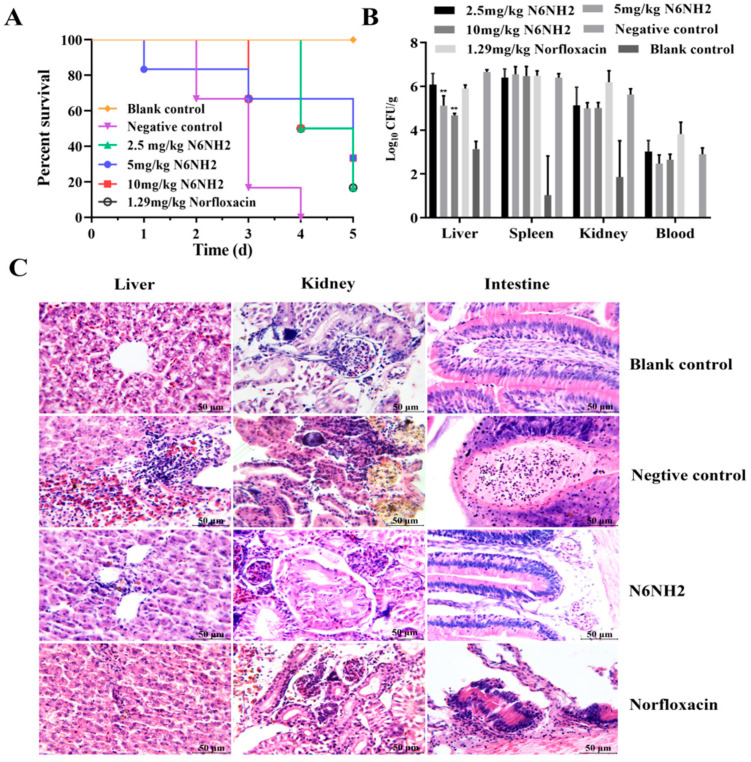
Efficacy of N6NH2 in vivo. (**A**) Survival of infectious *O. niloticus* treated with N6NH2 and norfloxacin. Fish (6 fish/group) were intraperitoneally injected with *E. tarda* (1.0 × 10^9^ CFU/mL), followed by injection with N6NH2 or norfloxacin at 0.5 h. Survival was recorded for 5 d. (**B**) Bacterial loads in the organs (including kidney, liver, spleen and blood). The challenge and treatment were performed as described above (10 fish/group). *E. tarda* was counted at 24 h after treatment with the N6NH2 or norfloxacin. (**C**) Effects of N6NH2 (10 mg/kg) or norfloxacin on the kidney, liver and intestine injuries induced by *E. tarda*. The kidney, liver and intestine specimens were collected from sacrificed fish. The sections were stained with hematoxylin and eosin and observed by a light microscope. All data were analyzed with one-way ANOVA, and data are means ± SD (n = 3). *p*-values < 0.05 were considered significant. **, *p* < 0.01.

As shown in Figure 6A, in the untreated group, the fish challenged with *E. tarda* began to die 12 h after inoculation, and all fish died within 3 d; in the blank control group, all fish survived. After treatment with 2.5 mg/kg N6NH2 or 1.29 mg/kg norfloxacin, the survival rate of fish was 16.7% within 2 d, but the survival rate of the fish treated with N6NH2 was higher than that in the norfloxacin group at 3 d. After 1 d treatment with peptides, the survival rate of fish treated with 5 mg/kg and 10 mg/kg N6NH2 was 100% and 80%, respectively, and both were 33.3% at 5 d. The main symptoms of the diseased fish treated with peptide were mostly abdominal distension, with some bulging eyes, varying degrees of bleeding on the body surface (including the fin, fin base, gill cover and mouth) (Supplementary Figure S3), visceromegaly of the spleen and kidney, and peritoneal effusion on autopsy. In contrast, the survival rate of fish treated with 1.29 mg/kg norfloxacin was 16.7%. This result indicates that one dose of N6NH2 can better protect *O. niloticus* from infection by *E. tarda* (Figure 6A) than norfloxacin. Meanwhile, N6NH2 may be used as a new strategy for the control of bacterial infection in fish due to its very low cytotoxicity, as mentioned above.

#### 2.7.3. Inhibition of Bacterial Translocation

To detect whether *E. tarda* is transferred from the abdominal cavity to other organs, blood, spleen, kidney and liver were collected and homogenized at 24 h after treatment with N6NH2 or norfloxacin. As shown in Figure 6B, after treatment with norfloxacin, the number of bacteria did not decrease in any organs except the liver, indicating its very low activity or no activity against *E. tarda*.

After treatment with 5 mg/kg and 10 mg/kg of N6NH2, *E. tarda* cells were significantly reduced by 23–30% in the liver. In 2.5–10 mg/kg N6NH2-treated groups, the bacterial burden in the kidney and blood was decreased by 8.7–11.2% and 8.6–15.2%, respectively (Figure 6B). This indicates that one dose of N6NH2 has some activity against *E. tarda*, but norfloxacin lacks antibacterial activity in organs.

#### 2.7.4. Alleviation of the Organ Injury

*E. tarda* infection significantly affected the histological structures of various organs of *O. niloticus.* As shown in Figure 6C, in the blank control group, it appears to normal structures of the liver lobule, and no degeneration and necrosis occurred in liver cells. The structures of glomeruli, renal tubules and colonic mucosa are also normal, with the epithelial cells arranging neatly. In the untreated fish, local lymphocytes and many eosinophil aggregations, disordered hepatocyte cords or even hepatocyte degeneration and necrosis were observed. After treatment with N6NH2, the damaged livers, kidneys and intestines of the infected fish apparently recovered and no obvious pathological symptoms occurred in livers and intestines. In the fish treated with norfloxacin, obvious pathological changes were found in organs, including hyperemia, inflammatory cells and necrotic and exfoliated mucosal epithelial cells (Figure 6C and Supplementary Figure S4). The results suggest that the therapeutic efficiency of a single dose N6NH2 in infectious fish is better than that of norfloxacin (Figure 6C).

#### 2.7.5. Regulation of the mRNA Levels of Immune-Related Genes

**Figure 7:**
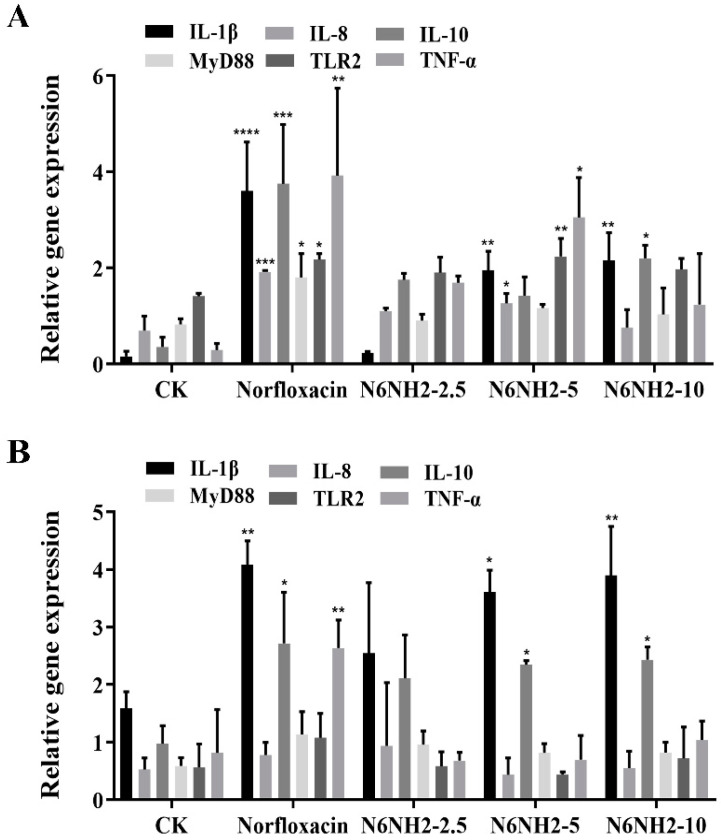
Effects of N6NH2 on cytokines. (**A**) The relative mRNA levels of IL-1β, IL-8, IL-10, MyD88, TLR-2 and TNF-α in the liver of *O. niloticus* after *E. tarda* infection. (**B**) The relative mRNA levels of IL-1β, IL-8, IL-10, MyD88, TLR-2 and TNF-α in the kidney of *O. niloticus* after *E. tarda* infection. All data were analyzed with one-way ANOVA, and data are means ± SD (*n* = 3). *p*-values of < 0.05 were considered significant. *, *p* < 0.05; **, *p* < 0.01; ***, *p* < 0.001; ****, *p* < 0.0001.

Compared with the control group, after treatment with N6NH2 or antibiotic, the mRNA levels of immune-related genes of the liver and kidney were upregulated in fish. N6NH2 downregulated the proinflammatory factors (interleukin-1β (IL-1β), interleukin-8 (IL-8) and tumor necrosis factor-α (TNF-α)) and upregulated the anti-inflammatory factor (interleukin-10 (IL-10)) in a concentration-dependent manner. After treatment with 10 mg/kg N6NH2, the mRNA levels of IL-10 were significantly increased by 1.84-fold in the liver, and the mRNA levels of IL-8 and TNF-α were reduced by 0.34–1.16-fold and 0.45–2.68-fold, respectively, which are superior to norfloxacin (Figure 7A). In the kidney, N6NH2 mainly upregulated the mRNA levels of pro-inflammatory factor (IL-1β) and anti-inflammatory factor (IL-10); compared with norfloxacin, N6NH2 downregulated the mRNA level of TNF-α gene (Figure 7B). N6NH2 had no significant effect on the mRNA levels of myeloid differentiation factor 88 (MyD88) and toll-like receptor-2 (TLR-2) genes, which are related to TLRs-nuclear factor-κB (NFκB) signaling pathway. These results thus indicate that N6NH2 significantly decreases the mRNA levels of proinflammatory cytokines and increases in the mRNA levels of anti-inflammatory cytokines in the liver and kidney compared with the antibiotic group.

## 3. Discussion

*E. tarda* is a fatal pathogen and can cause serious systemic infections and high mortality in fish [1]. Norfloxacin is one of the commonly used antibiotics for the treatment of fish Edwardsiellosis in aquatic products, and it has been used as a first-line fluoroquinolone antibiotic [20]. However, due to the emergence of resistant bacteria and potentially serious adverse effects on the musculoskeletal system and peripheral and central nervous system, the use of quinolones has been largely limited in food-animals in China since 2016 [20,26]. At present, there is a serious lack of new antibacterial agents to resolve the growing threat of antibiotic resistance. A few marine peptides—arenicin and its derivatives from *A. marina*—are short peptides endowed with a broad range of antibacterial activity against Gram-negative bacteria (including *E. coli*, *S. pullorum*, *S. enteritidis* and *P. aeruginosa*) and fungi (such as *Candida albicans*) and are less prone to trigger bacterial resistance [16,17,18]. In this study, we investigated the antibacterial activity of marine peptide-N6 and its amidated derivative-N6NH2 against *E. tarda* in vitro and in *O. niloticus* for the first time.

It has been suggested that an increase in the positive charges of AMPs favors electrostatic interactions between peptide segments and bacterial surfaces, followed by the introduction of hydrophobic portions into the bimolecular layer and thereby can improve antimicrobial activity against Gram-negative and Gram-positive bacteria, including *E. coli*, *A. pseudomonas* and *S. aureus* [27,28,29]. The positive charges of AMPs include a contribution from positively charged residues (Lys, His and Arg) and from the C-terminal amide group [30]. Nearly all AMPs can form amphipathic structures when they interact with the bacterial cell membranes [31]. There is evidence to suggest that the C-terminal amidation of some AMPs such as peptidyl-glycylleucine-carboxyamide (PGLa), human neutrophil α-defensin 2 (HNP2), tritrpticin and modelin-5 can enhance antimicrobial activity due to increased positive charges, which may change some physiochemical property such as amphiphilicity that affects their interaction with the bacterial cell membrane and hence improves the efficacy of their antimicrobial action [32,33,34,35,36]. Amidation has been found to be key with respect to the lytic activity of the peptides against pathogenic bacteria [36,37]. In our work, N6NH2 (with MICs of 0.65–1.29 µM) displayed more potent antibacterial activity against aquatic pathogens—both *E. tarda* and *A. veronii*—than N6 (the MICs of 3.2 µM) (Table 1), suggesting the functional charge contribution of the C-terminal amide [21,34,36].

A few studies have suggested that the secondary structure of AMPs may be affected by the C-terminal amidation in different solution conditions such as SDS and TFE [21,23,38]. In this study, N6 and N6NH2 were largely structured in ddH_2_O, SDS and TFE environments, with a variable number of β-strands (15.17–30.45%) and random coils (35.36–42.76%) and with relatively few helical domains (8.35–8.9%) (Figure 1B–D), which may be related to amidation of the C-terminus of peptide. Amidation may interfere with the intramolecular hydrogen bonds (HBs) in peptides and the HBs between solvent molecules and peptides. Therefore, the driving force to form β-sheet structures can be changed [23]. Both N6 and N6NH2 belong to β-sheet amphipathic peptides and may be organized to create both polar and nonpolar surfaces, which facilitate perturbing the bacterial membrane by insertion of the peptide hydrophobic face into the bacterial lipid bilayer, and thereby lead to the formation of transmembrane channels and leakage of the bacterial contents [31]. This effect was also seen in the outer membrane permeabilization and TEM results, in which the permeabilization of the outer membrane within 1 min (Figure 3A), the disappearance of the inner membrane and the ghost cells were present in most *E. tarda* cells after treatment with N6 and N6NH2, but not for norfloxacin (Figure 5), implying the cell membrane’s disruptive mechanism, the pore formation in the cell membrane induced by peptides and the leakage of bacterial materials [36]. Meanwhile, N6NH2 showed a higher ability to destroy the cell membrane of *E. tarda* than N6 (Figure 3A), indicating that C-terminal amidation can play a key role in membrane disruption by promoting peptide aggregation and membrane permeabilization by inverting the C-terminal charge [21,39].

Although both N6 and N6NH2 adopted low levels of α-helix in SDS solution (<11%), in the presence of *E. coli* LPS, N6NH2 had a higher ability than N6 to bind to LPS (Figure 4D). This is in line with the previous study in which the C-terminal amidation of β-sheet indolicidin could promote activity by facilitating better LPS binding and self-promoted uptake across the outer membrane [36]. Coupled with more bacterial outer membrane permeabilization at low concentrations (1 × and 2 × MIC) (Figure 3A), N6NH2 bound with higher affinity to bacterial genomic DNA than N6 (Figure 4A–C). The C-terminal amidation of N6 increased the cationic charge, which can promote the translocation of the peptide on the membrane of bacterial cells [22]. Additionally, positively charged residues (such as Arg) in N6 and N6NH2 may bind to the phosphate backbone of electronegative DNA, which is consistent with previous studies [25,40]. Further support for DNA-peptide binding via intercalation was given through the competitive binding assay of EB with bacterial DNA against N6 and N6NH2 (Figure 4B); with the addition of peptides, the characteristic fluorescence band of the DNA-peptide complex decreased gradually, indicating that some of the peptide molecules intercalated into the DNA base pairs instead of EB [25]. DNA-EB was replaced gradually by DNA-N6 and DNA-N6NH2. Meanwhile, both DNA-N6 and DNA-N6NH2 were replaced gradually by DNA-EB (Figure 4C), indicating a state of equilibrium between two complex systems of DNA-peptide and DNA-EB. These results support the suggestion that both N6 and N6NH2 can penetrate *E. tarda* cells by the membrane- and genomic DNA-disruptive mechanisms. Therefore, the bactericidal mechanism of N6 and N6NH2 may be similar to that of buforin II (BF2), which uses an uncommon mechanism to kill bacteria without causing cell lysis and is able to enter lipid vesicles through the bacterial membrane. After entering the cell, they bound to bacterial genomic DNA in an intercalating manner [40].

Although AMPs possess a wide spectrum of antimicrobial activity, their unfavorable properties, including low stability toward proteolytic degradation in the gastrointestinal tract and high toxicity toward eukaryotic cells, limit their application in vivo, which is of utmost importance for the administration of the drugs to be meaningful [31]. The chemical modification of N-/C-terminal amino or carboxylic acid functionalities, including amidation, acetylation or deamination, is usually used to inhibit enzymatic degradation and improve stability [31,41]. This modification advantage is that the peptide retains most of its unaltered structures, which hopefully will result in an unaffected bacterial activity [42]. It has also been demonstrated that C-terminal amidation can prevent the enzymatic degradation of AMPs such as cecropin [43]. In this study, N6NH2 had higher stability toward trypsin and lower hemolysis than N6 (Figure 2E,G) because the amidated version may prevent the action of exopeptidases [36]. Noticeably, the effect of N6 on *E. tarda* was more rapid and more profound than that of N6NH2 in bactericidal kinetics (Figure 2A,B), which needs more study in the future. Generally, N6NH2 has better activity, stability and biocompatibility than N6, indicating its great potential for application in the treatment of infectious diseases caused by *E. tarda*.

In the model of *O. niloticus* peritonitis, 2.5–10 mg/kg N6NH2 displayed higher therapeutic efficacy than 1.29 mg/kg norfloxacin after one-time administration, including enhanced survival rate of fish (33.3%), reduced the bacterial burden in the organs (8.6–30%) and alleviated the pathogenic injury of liver, intestine, and kidney (Figure 6). However, dose-independent effects of N6NH2 were observed; possible reasons for this are as follows: (i) the dosages of 2.5–10 mg/kg and one-shot protection of N6NH2 may not be within the sensitive range and effective administration times; (ii) high dosages of N6NH2 may cause toxicity risk and deny the positive effects [44]. Moreover, N6NH2 significantly induced an immune response by regulating the mRNA levels of IL-1β, IL-8, IL-10 and TNF-α in the liver and kidney in *O. niloticus* infected with *E. tarda* (Figure 7A,B), indicating the antibacterial and immunomodulatory activity of N6NH2. In previous studies, AMPs-human LL37 and GRN-41 in *Mozambican tilapia* upregulated the mRNA levels of immune-related genes such as TNF, IL-8, IL-1β, IL-6 and IL-10 [45,46]. This immunomodulatory activity of AMPs may be attributed to interleukins, interferons and tumor necrosis factors, which can play a vital role in fish immune response [47].

## 4. Materials and Methods

### 4.1. Bacterial Strains

The strains of *E. tarda* and *A. veronii* were clinically isolated from the diseased flounders in our laboratory. *S. hyicus* 437-2 and 15095 strains were isolated from infected pigs in Tianjin Animal Husbandry and Veterinary Research Institute. *S. typhimurium* ATCC14028 and *S. aureus* (ATCC43300, ATCC546 and ATCC25923) were from American Type Culture Collection (ATCC). Other *S. typhimurium* and *E. coli* strains were purchased from China Veterinary Culture Collection Center (CVCC). *P. aeruginosa* CICC21630 was obtained from China Center of Industrial Culture Collection (CICC).

### 4.2. Identification and Antibiotic Susceptibility Analysis of E. tarda

Bacterial genomic DNA was extracted from isolated *E. tarda* strain according to the instructions of TINAamp Bacteria DNA extraction kit. PCR amplification primers were designed according to the sequences of *E, tarda* 16S rRNA (16SF: 5′-AGAGTTTGATCCTGGCTCAG-3; 16SR: 5′-GGTTACCTTGTTACGACTT-3′) and DNA helicase B subunit protein gene (*gyr*B) (gyrF: 5′-GAAGTCATCATGACCGTTCTGCA-3′; gyrR: 5′-AGCAGGGTACGGATGTGCGAGCC-3′). All primers were synthesized by Sangon Biotech (Shanghai) Co., Ltd., (Shanghai, China). Using the extracted DNA as a template, PCR reactions were performed under the conditions of pre-denaturation at 98 °C for 3 min, 30 cycles of denaturation at 98 °C for 10 s, annealing at 55 °C for 10 s and extension for 1 min at 72 °C, and a final 7 min extension step at 72 °C. The PCR products were determined by 0.7% agarose gel electrophoresis and sequenced by Sangon Biotech (Shanghai) Co., Ltd.

The susceptibility of *E. tarda* to antibiotics was determined by the Kirby-Bauer disk diffusion method according to Clinical Laboratory Standards Institute (CLSI) [48]. *E. tarda* was cultured in TSB medium (10 mL) at 28 °C overnight. The MHA petri dish plates were seeded with a 250 μL bacterial solution (OD_600nm_ = 1.0). Antibiotic discs were placed onto the agar surface, and the plates were incubated at 37 °C for 18 h. The diameter of the bacterial inhibition zone was measured, and the results were determined according to the standard of CLSI.

### 4.3. Properties and Structural Analysis of N6 and N6NH2

The physicochemical properties (including MW, PI, net charge, hydrophobicity, and instability/aliphatic index) of peptides were calculated by ProtParam (https://web.expasy.org/protparam/ and https://pepcalc.com/). The peptide structures were predicated by the I-TASSER sever (http://zhanglab.ccmb.med.umich.edu/I-tasser).

CD spectroscopy was used to study the secondary structure of peptides [40]. The CD spectra of N6 and N6NH2 were measured by a MOS-450 spectral polarizer (Bio-Logic, Grenoble, France), a test tube using 1.0 mm wavelength. The peptide was dissolved in ddH_2_O, 20 mM SDS or 50% TFE solution, mimicking the cell membrane environment of bacteria or mammals. The spectra of the polypeptides were recorded from 180 nm to 260 nm at 25 °C at a scanning speed of 100 nm/min, step length resolution of 2.0 nm and integration time of 2 s. CDNN software is used to analyze the data.

### 4.4. Antimicrobial Activity and Time–Kill Curves of N6 and N6NH2

According to the CLSI guidelines, the MIC value was determined by the broth microdilution method [48]. The bacterial strains were cultured in Mueller–Hinton Broth (MHB) medium at 28 °C until the mid-log phase (OD_600nm_ of 1). Bacteria were diluted to 1 × 10^5^ CFU/mL and added into 96-well plates, followed by the addition of serial dilutions of peptides (from 0.0625 to 128 μg/mL). The plates were incubated at 37 °C for 18–24 h. The MIC value was determined as the lowest peptide concentration at which no bacterial growth was observed. The MBC of peptides or norfloxacin against *E. tarda* (>99.9% killing rate) was measured by subculturing the broths used for MIC determination onto fresh agar plates. All tests were conducted in triplicate.

Time–kill curves for N6 and N6NH2 were determined against *E. tarda* [20]. In brief, the mid-log phase bacterial cells (10^5^ CFU/mL) were mixed with different concentrations of peptides (1 × and 2 × and 4 × MIC) and cultured at 28 °C (200 rpm). Samples of 100 μL were taken from the mixture at intervals of 2 h, continuously diluted, and counted on the plate. The cells treated with norfloxacin (2 × MIC) were used as the positive control, and the cells without treatment were used as the blank control.

### 4.5. Stability of N6 and N6NH2 toward Temperature, pH and Proteases

To evaluate the thermal stability of peptides, N6 and N6NH2 were incubated for 1 h at 4, 20, 40, 60, 80 and 100 °C. To measure the pH stability, peptides were incubated for 3 h in 100 mM glycine-HCl buffer (pH 2.0), sodium acetate buffer (pH 4.0), sodium phosphate buffer (pH 6.0), Tris-HCl buffer (pH 8.0) or glycine-NaOH buffer (pH 10.0). In addition, peptides were incubated for 4 h at 37 °C in pepsin (3,000 U/mg, pH 2.0) and trypsin (250 U/mg, pH 8.0) (10:1, *w/w*) solutions, respectively. The final concentrations of N6, N6NH2 and norfloxacin were 128 μg/mL, 64 μg/mL, and 10 μg/mL, respectively. After treatment, 30 μL of solutions were taken out to determine the antibacterial activity of peptides against *E. tarda* by the inhibition zone method [49]. The untreated peptides were used as the control. All tests were conducted in triplicate.

### 4.6. Cytotoxicity and Hemolysis of N6 and N6NH2

The colorimetric MTT assay was used to determine the effect of peptides on the viability of murine peritoneal RAW 264.7 macrophage cells [50]. Macrophage cells were added into 96-well plates (5 × 10^3^ cells/well) and incubated for 24 h at 37 °C (5% (*v*/*v*) CO_2_/air). A series of peptide solutions were added into the cells and incubated for 48 h. The untreated cells were used as the control (A_control_). The MTT solution was added into plates, incubated for 4 h, and then removed from plates. Dimethyl sulfoxide (DMSO) was then added into plates and the absorbance was measured at 570 nm in a spectrophotometer. The cell viability was calculated using the following formula: Survival rate (%) = (A_peptide_ − A_control_)/A_control_ × 100.

In the hemolysis assay, the activity of peptides was evaluated by determining the amount of hemoglobin released in fresh mouse erythrocytes [17]. In brief, the blood cells were washed three times with 10 mM PBS (pH 7.4) and centrifuged at 1000 *g* for 10 min at room temperature. One hundred microliters of erythrocyte solution (8%, *v/v*) was mixed with 100 μL of a series of peptide solutions and incubated for 1 h at 37 °C. The mixture was then centrifuged at 1000× *g* for 5 min, and the absorbance of supernatants was measured at 540 nm. For the positive (A_100_) and control (A_0_), 0.1% Triton X-100 and PBS were used, respectively. Three replicates were performed for each condition. The hemolysis percentages of peptides were calculated by the following equation: Hemolysis (%) = [A_peptide_ − A_0_)/(A_100_ − A_0_)] × 100.

### 4.7. Antimicrobial Mechanism of N6 and N6NH2 against E. tarda

#### 4.7.1. Effects of Peptides on the Outer Membrane and Inner Membrane

The effect of peptides on the bacterial outer membrane of *E. tarda* was by using the fluorescent dye NPN assay [51]. *E. tarda* cells at mid-log phase were collected by centrifugation, washed twice, and suspended in HEPES buffer (pH 7.4). Cell suspensions (OD_600nm_ of 0.4) and NPN solutions (10 μM) were added into 96-well black plates, followed by the addition of peptide solutions (1 ×, 2 × and 4 × MIC). Fluorescence intensity was recorded at an excitation/emission spectrum of 328/438 nm until no further increase was observed with a microplate reader. The cells treated with PBS and norfloxacin were used as the negative and positive control, respectively.

To assess the effect of peptides on the inner membrane, mid-log phase *E. tarda* cells (1 × 10^8^ CFU/mL) were incubated with or without peptides (1×, 2×, and 4× MIC) for 5, 15, 30, and 120 min, respectively. After washing twice with PBS, the cells were incubated with PI (50 μg/mL) for 15 min and analyzed using a FACS Calibur Flow Cytometer (BD, San Jose, CA, USA) [52].

#### 4.7.2. Interaction with DNA

The genomic DNA of *E. tarda* or *O. niloticus* was incubated for 10 min at room temperature with different concentrations of N6 and N6NH2 in binding buffer (5% glycerol, 10 mM Tris-HCl, pH 8.0, 20 mM KCl, 1 mM EDTA, 1 mM dithiothreitol and 50 mg/mL BSA). The mass ratios of peptides and DNA were 0, 0.5, 1, 2, 4, 6, 8, and 10, respectively. The migration of DNA was evaluated by electrophoresis on a 0.7% agarose gel [51,53].

#### 4.7.3. Competitive Binding of Genomic DNA with EB

*E. tarda* genomic DNA was dissolved in TE buffer to a concentration of 100 μg/mL; 1.5 μL of EB (100 μg/mL) solution was mixed with 100 μL of DNA solution and then added into 96-well plates. The DNA-EB mixture was incubated in the dark at 37 °C for 10 min. Varying concentrations of peptides (0, 40, 80, and 160 μg/mL, 100 μL) were then added to the DNA-EB solution (100 μL), and the fluorescence spectra were measured for each test solution after 30 min of incubation at 37 °C in the dark. Infinite M200 PRO was used for sample determination at 535 nm, and the spectra were recorded from 550 to 750 nm [25].

To determine the competitive binding of EB against peptides with bacterial DNA, 100 μL of DNA (50 μg/mL) solution was mixed with 100 μL of peptide solution (80 μg/mL) and incubated at 37 °C for 10 min. Then aliquots of stock solution of EB (0, 25, 50, and 100 μg/mL, 1.5 μL) were added to the fixed DNA-peptide solution and incubated for 30 min at 37 °C in the dark. The fluorescence spectra were measured as described above.

#### 4.7.4. Binding to LPS

The BC probe was used to determine the ability of peptides to bind to LPS [54]. A total of 0.5 µM LPS was incubated with 5 μM BC in 50 µM Tris buffer (pH 7.4) for 4 h at 37 °C. Subsequently, N6 or N6NH2 was added into the mixture, and the final concentrations of peptides ranged from 0.049 μM to 50 μM. Ampicillin was used as the negative control. Changes in fluorescence were measured by fluorescence spectrophotometry at room temperature (excitation/ emission, 580/620 nm).

### 4.8. Effects of N6 and N6NH2 on the Morphology of E. tarda

Mid-log phase *E. tarda* cells (1 × 10^8^ CFU/mL) were incubated with 4 × MIC N6 or N6NH2 for 2 h at 37 °C. The bacterial cells were then centrifuged, washed three times with 0.1 M PBS (pH 7.2) and fixed overnight at 4 °C with 2.5% glutaraldehyde. After washing twice with PBS, the cells were post-fixed with 1% osmium tetroxide (OsO_4_) for 2 h, dehydrated in a graded ethanol series (from 50% to 100%) for 15 min each time and dried by CO_2_. The cell samples were sputtered with gold-palladium and observed on a QUANTA200 electron microscopy (FEI, Philips, The Netherlands) [53].

The bacterial cells were treated with peptides as described above, fixed with 2.5% glutaraldehyde at 4 °C overnight and post-fixed with 1% OsO_4_ for 1 h. After washing three times with PBS, the cells were dehydrated with a graded acetone series (50%, 70%, 85%, 95% and 100%, for 7 min each time) and immersed in mixtures of acetone and resin (3:1, 1:1 and 1:3, 40 min each time). The samples were infiltrated with a pure epoxy resin overnight, embedded in capsules containing embedding medium and polymerized at 45 °C for 3 h and at 65 °C for 24 h, respectively. Thin sections were cut using an ultramicrotome, stained with 1% uranyl acetate and examined by a JEM1400 (JEDL, Tokyo, Japan) [51].

### 4.9. In Vivo Experiments of N6NH2

All *O. niloticus* experiments were performed in accordance with the Animal Care and Use Committee of the Feed Research Institute of the Chinese Academy of Agricultural Sciences (CAAS), and protocols were approved by the Laboratory Animal Ethical Committee and its Inspection of the Feed Research Institute of CAAS (AEC-CAAS-20090609).

#### 4.9.1. Efficacy of N6NH2

The fish (*O. niloticus*, Gift strain, 20 g mean weight) were purchased from Beijing Fisheries Technology Extension Center, Hainan Station, China and were acclimated at 25 ± 1 °C. The fish were fed twice daily with commercial pellets for two weeks before experiments.

Mid-log phase *E. tarda* was centrifugated, resuspended with PBS and diluted with PBS to different concentrations (1 × 10^7^, 5 × 10^7^, 1 × 10^8^, 5 × 10^8^, 1 × 10^9^ CFU/mL). A total of 30 *O. niloticus* were randomly divided into 5 groups (6 fish/group). Fish were intraperitoneally injected with *E. tarda* (200 μL/fish), and the survival rate of fish was recorded for 6 d to obtain an LC_50_ and LC_100_ of *E. tarda*.

*O. niloticus* (6 fish/group) were intraperitoneally injected with LC_100_ of *E. tarda*, followed 30 min later by 200 µL N6NH2 (2.5, 5, and 10 mg/kg, respectively). Norfloxacin was used as a positive control. Fish injected with only bacteria or saline served as negative (untreated) or blank (unchallenged) controls, respectively. Fish treated with norfloxacin (1.29 mg/kg) at the equimolar highest concentration of peptide were used as a positive control. The fish survival rate was recorded daily for 5 d. Two independent experiments were conducted, including pre-experiment and formal experiment.

#### 4.9.2. Inhibition of Bacterial Translocation

Sixty fish were randomly divided into 6 groups (10 fish/group). Among them, 5 groups were treated with N6NH2 (with doses of 2.5, 5, and 10 mg/kg) and norfloxacin (1.29 mg/kg), respectively. The remaining untreated group served as negative control. The fish injected with saline were used as the blank control. The challenge and treatment were performed as described above. The kidney, liver, spleen and blood samples (3 fish/group) were taken after 18 h and homogenized in sterile PBS for the CFU assay to evaluate *E. tarda* translocation. Other fish were used to analyze the effects on tissues and immune genes.

#### 4.9.3. Histopathological Changes of Tissues

The spleen, kidney, liver, intestine and brain samples (3 fish/group) were used for tissue section observation. After 24 h of fixation, all samples were dehydrated with 75–95% ethanol, embedded in paraffin and cut to 6 μm thin sections. Sections were stained with hematoxylin and eosin and observed by a light microscope.

#### 4.9.4. Effects on the Production of Immune-Related Genes

RNA was extracted from the liver, kidney and spleen samples of *O. niloticus* (3 fish/group) using Trizol reagent (Invitrogen Life Technologies, Carlsbad, CA, USA). cDNA was synthesized from 2 µg of total RNA using the RevertAid First Strand cDNA Synthesis Kit (Thermo Fisher, Waltham, MA, USA) with oligo dT primers following the manufacturer’s instructions. Real-time PCR was conducted on a real-time PCR system (Hongshi, Co.,Ltd., Shanghai, China) using the FastStart Universal SYBR Green 2 × qPCR Master mix (Roche, Basel, Switzerland). The primers of IL-1β, IL-8, IL-10, MyD88, TLR-2 and TNF-α are given in Supplementary Table S1. Each PCR was performed with triplicate samples and the cycling conditions were 10 min at 95 °C, 15 s at 95 °C and 60 s at 60 °C for 40 cycles. Then, the relative abundance of the target genes was calculated by using the 2^−ΔΔCt^ [55].

### 4.10. Statistical Analysis

GraphPad Prism 7.0 (GraphPad Software, LaJolla, CA, USA) was used for all statistical analyses, and *p* < 0.05 was statistically significant.

## 5. Conclusions

We investigated the bactericidal activity and mechanism of N6 and N6NH2 against *E. tarda* and primary application for *O. niloticus*. N6NH2 showed stronger antibacterial activity against *E. tarda*, lower hemolysis and higher stability toward trypsin than N6. The two peptides could destroy the outer membrane and bind to genomic DNA of *E. tarda*. N6NH2 better protected *O. niloticus* from infection caused by *E. tarda* than norfloxacin. Our results indicate that N6NH2 is a promising candidate for further research as a novel antimicrobial agent in aquatic animals.

## Figures and Tables

**Table 1:** Amino acid sequences and physiochemical properties of N6 and N6NH2.

Peptides	Amino Acid Sequence	Theoretical MW (Da)	Measured MW (Da)	PI	Charge	GRAVY	II	AI
N6	GFAWNVCVYRNGVRVCHRRAN	2477.85	2475.87	10.72	+4	−0.31	41.69	64.76
N6NH2	GFAWNVCVYRNGVRVCHRRAN-NH2	2476.85	2476.8	11.64	+5	−0.31	41.69	64.76

MW: molecular weight; PI: isoelectric point; GRAVY: grand average of hydropathicity II: instability index; AI: aliphatic index.

**Table 2:** The MIC values of N6 and N6NH2.

Species and Strains	N6NH2	N6 ^a^	Norfloxacin	N6NH2	N6 ^a^	Norfloxacin
	(μg/mL)			(μM)		
Gram-negative bacteria						
*Edwardsiella tarda*	3.2	6.4	0.5	1.29	2.58	1.57
*Aeromonas veronii*	1.6	6.4	2	0.646	2.58	6.26
*Escherichia coli* CVCC195	1.6	0.5	0.06	0.646	0.2	0.19
*E. coli* CVCC1515	0.8	1	0.06	0.323	0.4	0.19
*E. coli* CVCC25922	1.6	0.25	0.06	0.646	0.1	0.19
*E. coli* CVCCO157	1.6	0.5	0.06	0.646	0.2	0.19
*Salmonella typhimurium* CVCC533	1.6	1	0.25	0.646	0.4	0.78
*S. typhimurium* ATCC14028	1.6	2	0.06	0.646	0.8	0.19
*S. enteritidis* CVCC3377	0.8	0.25	0.06	0.323	0.1	0.19
*S. pullorum* CVCC1802	1.6	0.5	1	0.646	0.2	3.13
*S. pullorum* CVCC1789	3.2	0.5	0.13	1.29	0.2	0.41
*Pseudomonas aeruginosa* CICC21630	25.6	4	ND	10.34	1.6	ND
Gram-positive bacteria						
*Staphylococcus aureus* ATCC43300	6.4	16	1	2.58	6.46	3.13
*S. aureus* ATCC546	6.4	4	1	2.58	1.62	3.13
*S. aureus* ATCC25923	12.8	0.25	1	5.17	0.1	3.13
*S. hyicus* 437-2	12.8	64	16	5.17	25.8	50.1
*S. hyicus* 15095	3.2	16	4	1.29	6.46	12.5

ND: no data. No experiment with this strain. ^a^ Some data were from our previous study [19]. Data were representative of three independent experiments.

## Supplementary Materials

The following are available online at https://www.mdpi.com/1660-3397/18/12/650/s1: Figure S1. Interaction of bacterial genomic DNA (25–400 μg/mL) with N6 and N6NH2 (0.5 μg) or norfloxacin by a gel migration assay. Figure S2. Interaction of N6 and N6NH2 with genomic DNA from *O. niloticus* by a gel migration assay. Figure S3. Pathogenic symptoms of *E. tarda*-infected diseases occurred in dead tilapia. Figure S4. Effects of N6NH2 (2.5 and 5 mg/kg) on the kidney, liver and intestine injuries induced by *E. tarda*. Table S1: Primers used for real-time PCR analysis, Table S2: Susceptibility analysis of *E. tarda* against antibiotics.
