# Supplementary material for: Improved Stability and Activity of a Marine Peptide-N6NH2 against Edwardsiella tarda and Its Preliminary Application in Fish
Source: Mar Drugs. 2020 Dec 17;18(12):650. doi: 10.3390/md18120650 (PMC7766155; doi:10.3390/md18120650)
Supplement: Supplementary file 1 [file marinedrugs-18-00650-s001.pdf]

Supplementary Materials

Table S1. Primers used for real-time PCR analysis.

Genes	GeneBank No.	Primer sequences (5′→3′)	Fragment length (bp)
IL-1β	XM_019365844.2	F: TCTGGTAATCCACTCAAATAGGGA	73
		R: ACCTTGTGATGTAGTGTTGTGGT	
IL-8	XM_019353306.2	F: TGAGCAGATCTTGCATGTAGC	101
		R: GCTGCTGATCAAGAAGATTCACC	
IL-10	KP645180.1	F: TCAGCAAGATCCAACAAAGCC	137
		R: CAGGAGGACGGTCTGAGAAGT	
MyD88	NM_001311322.1	F: CCAACGCCAGCAAAGTTCTC	81
		R: AGGTCCACACAAAACCCCTG	
TLR2	XM_019360109.2	F: AATTGTGACCAAGACGGGACA	107
		R: GTTGACTGGTGAGCGACGA	
TNF-α	XM_003456260.4	F: GGACACTGGCCACTTACGAA	140
		R: GCCAACCTTCCCAAAACCAC	
β-actin	XM_003443127.5	F: CACCGTGCTGTCTGGAGGTA	122
		R: ATTTACGCTCAGGTGGGGCAAT	

Table S2. The susceptibility analysis of *E. tarda* against antibiotics.

Antibiotics	Disc content (μg)	Interpretation of zone diameters (mm)			Diameters of bacteriostatic zone (mm)	Sensibility
		R	I	S		
Streptomycin	10	≤11	12–14	≥15	29	S
Cefotaxime	30	≤14	15–22	≥23	31	S
Vancomycin	30	≤14	15–16	≥17	28	S
Chloromycetin	30	≤12	13–17	≥18	30	S
Norfloxacin	10	≤12	13–16	≥17	26	S
Erythromycin	15	≤13	14–22	≥23	41	S
Gentamicin	10	≤12	13–14	≥15	35	S
Tetracycline	30	≤14	15–18	≥19	27	S
Cefazolin	30	≤14	15–17	≥18	28	S
Ampicillin	10	≤13	14–16	≥17	24	S
Amoxicillin	20	≤13	14–17	≥18	12	R
Oxacillin	1	≤10	11–12	≥13	13	S
Ciprofloxacin	5	≤15	16–20	≥21	34	S
Bacitracin	0.04 U[a]	≤9	10	≥11	6	R
Lomefloxacin	10	≤18	19–21	≥22	31	S
Ofloxacin	5	≤12	13–15	≥16	33	S
Cefalotin	30	≤14	15–17	≥18	25	S
Florfenicol	30	≤12	13–17	≥18	28	S
Rifampicin	5	≤16	17–19	≥20	32	S
Polymyxin B	300	≤8	8–11	≥12	18	S
Doxycycline	30	≤10	11–13	≥14	24	S
Tobramycin	10	≤12	13–14	≥15	35	S
Clindamycin	2	≤14	15–20	≥21	25	S
Furazolidone	300	≤14	15–16	≥17	12	R
Macrodantin	300	≤14	15–16	≥17	30	S
Cefuroxime	30	≤14	15–22	≥23	30	S
Ceftriaxone	30	≤13	14–20	≥21	31	S

Note: R: resistance; S: sensitivity; I: intermediate. a: unit.

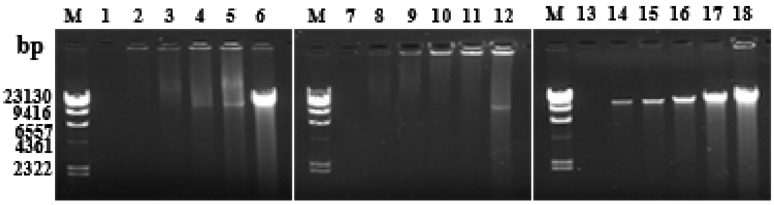

Figure S1. Interaction of bacterial genomic DNA (25–400 μg/mL) with N6 and N6NH2 (0.5 μg) or norfloxacin by a gel migration assay. M: DNA marker λDNA/HindIII; 1–6: the mass ratios of DNA/N6 were 0, 0.125, 0.25, 0.5, 1, and 2, respectively; 7–12: the mass ratios of DNA/N6NH2 were 0, 0.125, 0.25, 0.5, 1, and 2, respectively; 13–18: the mass ratios of DNA/norfloxacin were 0, 0.125, 0.25, 0.5, 1, and 2, respectively.

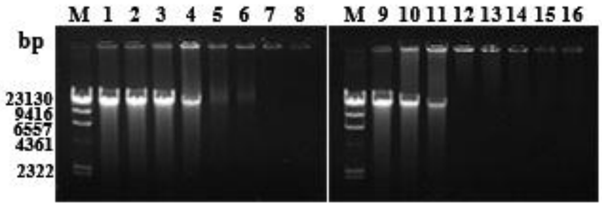

Figure S2. Interaction of N6 and N6NH2 with genomic DNA from *O. niloticus* by a gel migration assay. M: DNA marker λDNA/HindIII; 1–8: the mass ratios of N6/DNA were 0, 0.5, 1, 2, 4, 6, 8, and 10, respectively; 9–16: the mass ratios of N6NH2/DNA were 0, 0.5, 1, 2, 4, 6, 8, and 10, respectively.

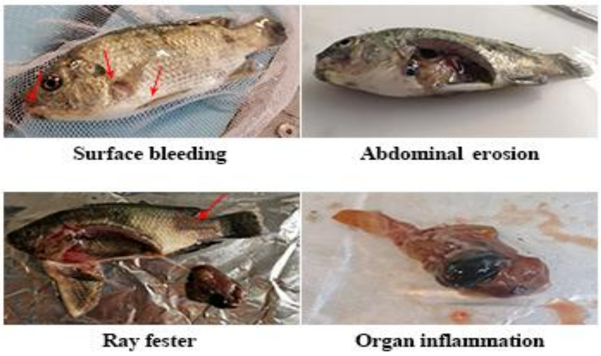

Figure S3. Pathogenic symptoms of *E. tarda*-infected diseases occurred in dead tilapia. It was observed that varying degrees of bleeding on the body surface (including the fin, fin base, gill cover, and mouth, respectively), with some bulging eyes, abdominal swelling or erosion in fish with ascites, and organ inflammation. Arrows indicate typical pathogenic symptoms.

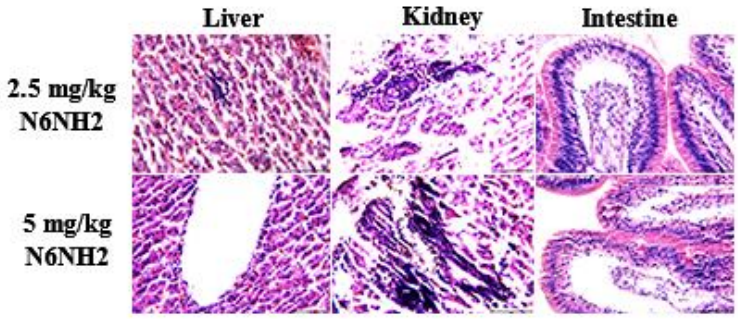

Figure S4. Effects of N6NH2 (2.5 and 5 mg/kg) on the kidney, liver and intestine injuries induced by *E. tarda*.
